# Host-Specific Response to HCV Infection in the Chimeric SCID-beige/Alb-uPA Mouse Model: Role of the Innate Antiviral Immune Response

**DOI:** 10.1371/journal.ppat.0020059

**Published:** 2006-06-23

**Authors:** Kathie-Anne Walters, Michael A Joyce, Jill C Thompson, Maria W Smith, Matthew M Yeh, Sean Proll, Lin-Fu Zhu, T. J Gao, Norman M Kneteman, D. Lorne Tyrrell, Michael G Katze

**Affiliations:** 1 Department of Microbiology, School of Medicine, University of Washington, Seattle, Washington, United States of America; 2 Department of Medical Microbiology and Immunology, University of Alberta, Edmonton, Alberta, Canada; 3 Department of Pathology, School of Medicine, University of Washington, Seattle, Washington, United States of America; 4 Department of Surgery, University of Alberta, Edmonton, Alberta, Canada; The Rockefeller University, United States of America

## Abstract

The severe combined immunodeficiency disorder (SCID)-beige/albumin (Alb)-urokinase plasminogen activator (uPA) mouse containing a human-mouse chimeric liver is currently the only small animal model capable of supporting hepatitis C virus (HCV) infection. This model was utilized to characterize the host transcriptional response to HCV infection. The purpose of these studies was to investigate the genetic component of the host response to HCV infection and also to distinguish virus-induced gene expression changes from adaptive HCV-specific immune-mediated effects. Gene expression profiles from HCV-infected mice were also compared to those from HCV-infected patients. Analyses of the gene expression data demonstrate that host factors regulate the response to HCV infection, including the nature of the innate antiviral immune response. They also indicate that HCV mediates gene expression changes, including regulation of lipid metabolism genes, which have the potential to be directly cytopathic, indicating that liver pathology may not be exclusively mediated by HCV-specific adaptive immune responses. This effect appears to be inversely related to the activation of the innate antiviral immune response. In summary, the nature of the initial interferon response to HCV infection may determine the extent of viral-mediated effects on host gene expression.

## Synopsis

The natural history of hepatitis C virus (HCV) infection is highly variable, and approximately 30% of chronically infected patients will develop progressive liver disease, including fibrosis, cirrhosis, and hepatocellular carcinoma (HCC). This high variability in HCV-associated liver disease, ranging from mild inflammation to rapidly progressive fibrosis, suggests that host factors play an important role in both infection outcome and viral pathogenesis. In the current study, the severe combined immunodeficiency disorder-beige/albumin-urokinase plasminogen activator mouse model was used to investigate how host-specific factors influence the host response to HCV infection. Cohorts of mice transplanted with hepatocytes from different donors were inoculated with a single source of HCV. Gene expression profiling was performed to characterize the host response to infection. The results indicate that host factors do contribute to the variation in host response to HCV infection, including the activation of innate antiviral signaling pathways. They also suggest that the nature of the innate antiviral immune response during the acute phase of infection may determine the extent of viral-mediated effects on host gene expression, including regulation of lipid metabolism genes and induction of stress-response genes. In addition, the presence of apoptotic hepatocytes in HCV-infected mice suggests that liver injury can occur in the absence of an adaptive HCV-specific immune response.

## Introduction

Hepatitis C virus (HCV) is a blood-borne pathogen belonging to the Flaviviridae family. There are over 170 million people worldwide chronically infected with HCV. The natural history of HCV infection is highly variable and approximately 30% of chronically infected patients will develop progressive liver disease, including fibrosis, cirrhosis, and hepatocellular carcinoma (HCC) [1]. Although exposure to HCV generally results in chronic infection, individuals can often be infected for decades with minimal liver damage, suggesting that the effect of HCV on hepatocyte function is extremely subtle. In addition, the high variability in HCV-associated liver disease, ranging from mild inflammation to rapidly progressive fibrosis, suggests that host factors play an important role in both infection outcome and viral pathogenesis. It is generally thought that the pathology associated with chronic HCV infection is mediated by an HCV-specific cell-mediated immune response [2]. The role of HCV replication, and subsequent virus-host interactions, in the pathology of chronic infection remains unclear. Several studies have attempted to probe the complexity of HCV-host interactions by performing global transcriptional profiling on liver biopsy samples from HCV-infected individuals and chimpanzees [3–9]. Not surprisingly, these studies have revealed substantial variation in the host response to infection. There are several possible contributing factors to this variation, including duration of infection, extent of liver disease, and viral factors including genotype and quasispecies diversity. This makes it difficult to assess the individual role that host factors play in this variation. In addition, these studies are complicated by the presence of an HCV-specific adaptive immune response, making it difficult to distinguish immune-mediated and viral-induced gene expression changes.

In the current study, the severe combined immunodeficiency disorder (SCID)-beige/albumin (Alb)-urokinase plasminogen activator (uPA) mouse model was used to investigate how host-specific factors influence the host response to HCV infection. These animals are derived by transplantation of normal human hepatocytes into SCID mice carrying a plasminogen activator transgene (Alb-uPA) [10–13]. The model has significant advantages over in vitro systems in that it represents an in vivo infection, all HCV proteins are expressed at biologically relevant levels, and infectious virions are assembled and released from hepatocytes. Together with the use of highly sensitive oligonucleotide microarray technology, this model provides several features that make it a unique system in which to study HCV-host interactions. First, because these animals are transplanted with hepatocytes from different donors, there is the opportunity to analyze host-specific responses to HCV. Second, both viral inoculum and duration of infection can be controlled, making it easier to interpret the cause of variation in the global gene expression profiles. Finally, the lack of an adaptive immune response in these animals makes it possible to distinguish viral-mediated from immune-mediated effects on host gene expression.

Cohorts of mice transplanted with hepatocytes from different donors were inoculated with a single source of HCV. Gene expression profiling of liver tissue was then performed to characterize the host response to infection. The results indicate that host factors do contribute to the variation in host response to HCV infection and that the nature of the initial innate antiviral immune response may determine the extent of viral-mediated effects on host gene expression.

## Results

### Host Factors Influence the Response to HCV Infection in the SCID-beige/Alb-uPA Mouse

Chronic HCV infection results in a highly variable course of liver disease, ranging from mild inflammation maintained over decades to rapidly progressive fibrosis, cirrhosis, and HCC. Even when patients are infected with the same genotype, severe liver disease occurs in only a subset of chronically infected individuals, suggesting that host factors likely play an important role in the progression of liver disease [14,15]. In order to investigate the contribution of host factors in the response to infection, cohorts of mice transplanted with hepatocytes from different donors were infected with a single source of HCV (+) patient serum (genotype 1a). At 4 to 7 wk after exposure to HCV, the animals were killed, and the human liver hepatocytes were isolated and characterized as outlined in Materials and Methods. Information regarding serum HCV levels, duration of infection, and purity of liver samples is shown in Table 1. Gene expression microarray experiments were then performed to characterize the host response to HCV infection. However, since liver samples from these animals typically had small percentages of contaminating mouse liver tissue (Table 1), the level of cross-hybridization of mouse liver mRNA to the human probes present on the microarray was first assessed [58]. The small set of genes identified (less than 2% of genes on the array) were either removed from subsequent analysis of gene expression data or changes in the expression of these genes were validated using human-specific quantitative real-time (RT)-PCR.

**Table 1:**
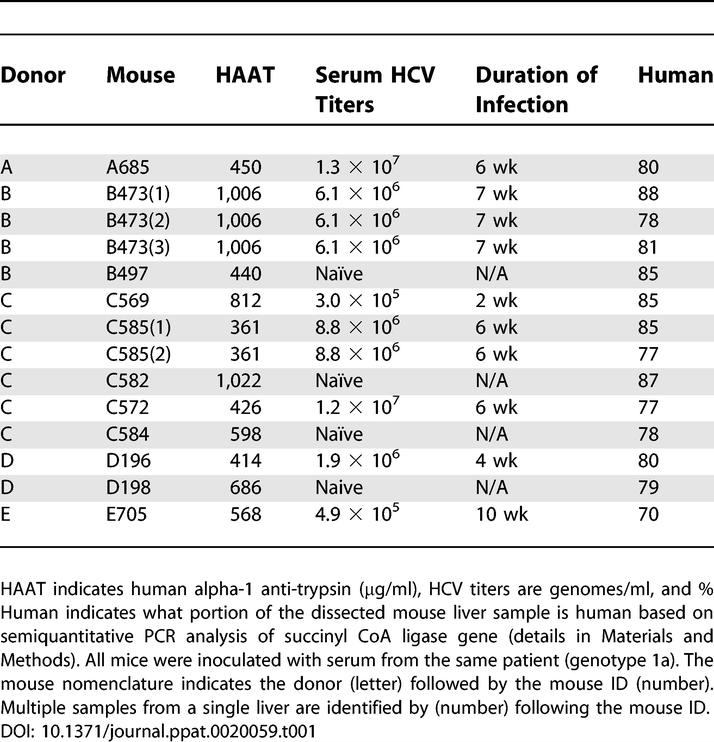
Characteristics of Mouse Liver Samples

For the microarray experiments, mRNA samples isolated from four HCV-infected animals containing hepatocytes from three different donors were compared to mRNA isolated from their donor-matched uninfected controls. Because each pair of mice contained hepatocytes from the same donor, changes in gene expression should mainly be induced by the HCV infection and independent of host variation. In general, the effect on host gene expression by HCV infection was not that extreme, with 390 genes showing a 2-fold or higher change in expression (*p* ≤ 0.05) in at least two experiments (Figure 1). This is similar to what is observed during acute HCV infection in chimpanzees [6]. The grouping of experiments by the clustering algorithm suggested that the effect on host gene expression was similar between mice containing the same donor hepatocytes. In contrast, the effect on host gene expression appeared quite different between mice containing different donor hepatocytes. The correlation coefficient for common differentially regulated genes was also higher between mice containing the same donor hepatocytes than between mice containing different hepatocytes (unpublished data). Since all animals were inoculated with the same HCV (+) serum, infected for similar lengths of time, and in general showed similar serum HCV RNA levels (Table 1), these results suggest that host-specific factors are influencing the response to HCV infection.

**Figure 1:**
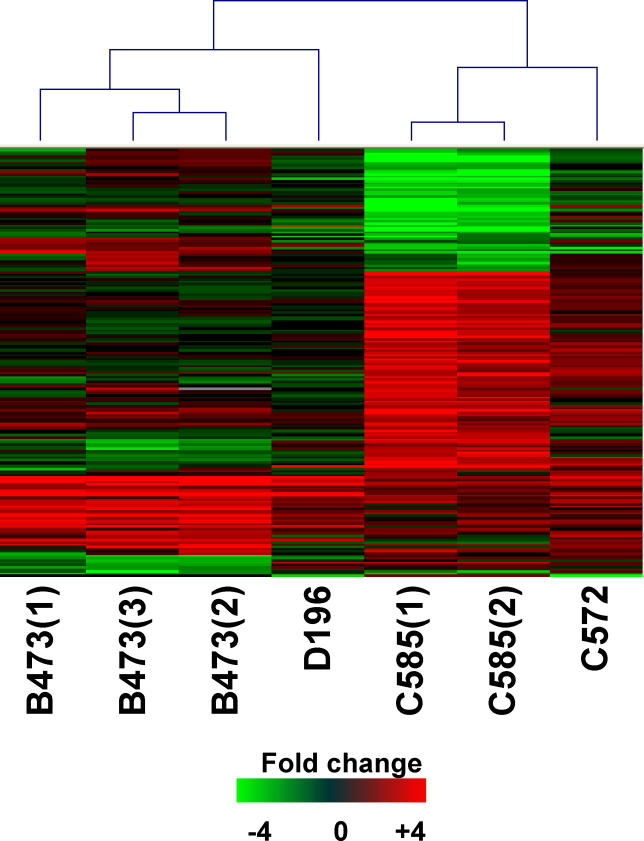
Global Gene Expression Analysis of HCV-Infected Mice Two-dimensional hierarchical clustering was done using Resolver System software with an agglomerative algorithm, average link heuristic criteria, and Cosine correlation metric. Each column represents gene expression data from an individual experiment (either individual HCV-infected mouse or individual liver sample) and the cluster represents genes that showed a greater than 2-fold change (*p* <0.05) in at least two experiments. Genes shown in red were up-regulated, genes shown in green were down-regulated, and genes shown in black indicate no change in expression in HCV-infected tissue relative to donor-matched uninfected tissue. Experiments using separate liver pieces from the same HCV-infected mouse used the same uninfected sample as the reference.

### HCV Infection in Mice Stimulates Innate Antiviral Signaling Pathways

Analysis of the expression of genes encoding proteins involved in interferon (IFN) signaling pathways and genes known to be transcriptionally regulated by IFN has shown that chronically infected HCV individuals show evidence of an IFN response [5,16]. Gene expression analysis of liver tissue from HCV-infected mice also demonstrated an up-regulation of many known IFN-stimulated genes (ISGs). These included key genes associated with the IFN-signaling pathway, such as STAT1, IRF9, OAS, Mx1, TLR3, major histocompatibility complex (MHC) class I, and PKR (Figure 2A). There was also an up-regulation of genes associated with the immunoproteasome, including PSME2, PSMB9, and USP18, which plays a role in antigen processing and presentation on MHC class I molecules. Many of the up-regulated ISGs in HCV-infected mice are also induced during the acute phase of HCV infection of chimps as well as chronically infected patients [5–7,9].

**Figure 2:**
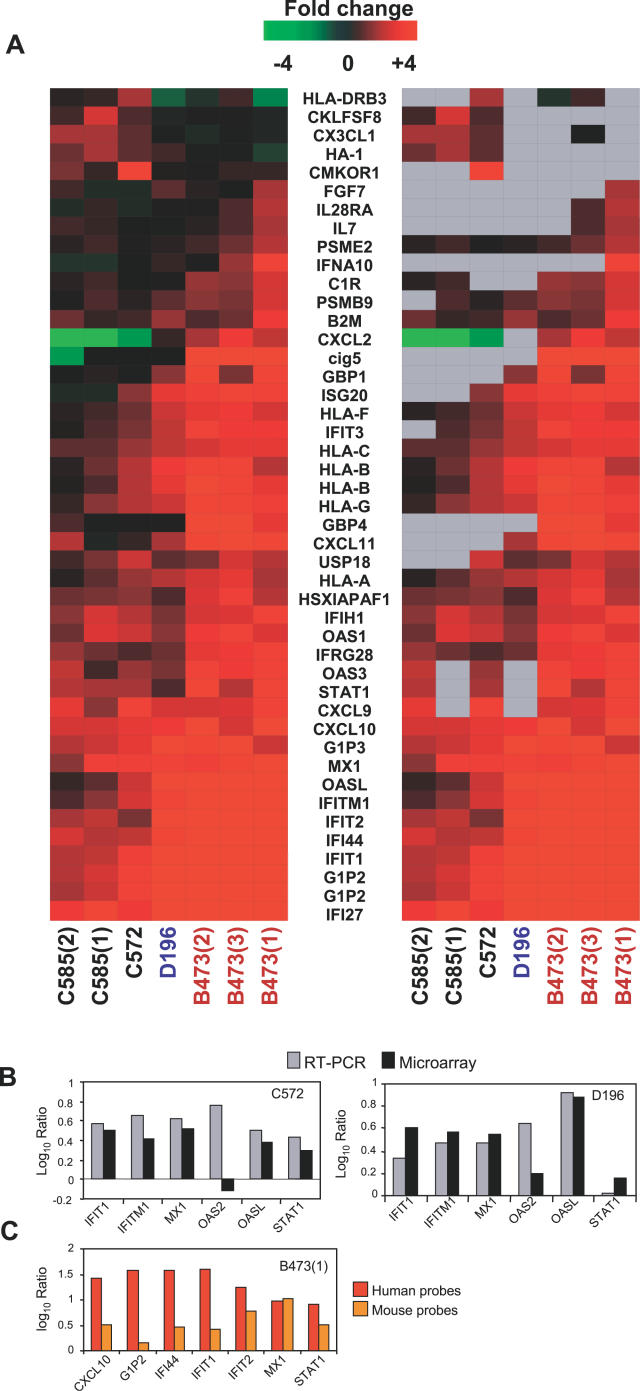
Expression of IFN-Inducible Genes in Human Liver Tissue from HCV-Infected Mice (A) Two-dimensional hierarchical clustering was done using Resolver System software with an agglomerative algorithm, complete link heuristic criteria, and Euclidean correlation metric. Each column represents gene expression data from an individual experiment (either individual HCV-infected mouse or individual liver sample). Genes were selected as at least 2-fold regulated (*p* < 0.05) in at least one of seven experiments. In the left panel, genes shown in red are up-regulated and genes shown in green are down-regulated in HCV-infected tissue relative to donor-matched uninfected tissue, while black indicates no change in gene expression. In the right panel, genes whose regulation showed *p* > 0.05 are shown in gray. (B) RT-PCR validation of the expression array data performed for six IFN-inducible genes in a subset of HCV-infected mice. Data are shown as log_10_ ratio and reflect the difference in expression between donor-matched HCV-infected and naïve liver tissue. (C) Expression of IFN-regulated genes in human and mouse liver tissue. Comparison of gene expression levels of IFN-regulated genes by RT-PCR analysis used either human- or mouse-specific probes. Data are shown as log_10_ ratio and reflect the difference in expression between donor-matched HCV-infected and naïve liver tissue.

The up-regulation of ISGs was seen when the HCV-infected liver tissue was compared with either donor-matched uninfected tissue (Figure 2A) or a reference pool of normal, uninfected human tissue (data not shown). In contrast, liver tissue from uninfected mice did not show an up-regulation of these genes, demonstrating that the induction of IFN-inducible genes is specific to HCV and not the result of the isolation and transplantation of the human hepatocytes into the mice (unpublished data). Quantitative PCR analysis of a number of the IFN-regulated genes demonstrated a good correlation with the gene expression data from the microarrays (Figure 2B), although the ratios calculated from RT-PCR generally exceeded those obtained using microarrays. Interestingly, the use of mouse-specific probes for RT-PCR analysis also demonstrated an induction of the IFN response in the mouse hepatocytes, although in general at a much lower level of induction than that seen in the human hepatocytes from the same mouse (Figure 2C).

Although all HCV-infected mice showed evidence of induction of innate antiviral signaling pathways, the response was variable between mice, most notably between mice containing hepatocytes from different donors (Figure 2A). This is particularly interesting as the mice were all inoculated with equal amounts of the same human serum containing HCV genotype 1a. The IFN response appeared to be independent of viral factors including serum HCV titers and length of infection. For example, animals C585 and B473 had comparable serum HCV titers and lengths of infection, yet mouse B473 showed a much stronger induction of IFN-regulated genes. In addition, mouse D196 had only been infected for approximately 4 wk, yet showed a very strong IFN response, as indicated by the number and level of induction of ISGs. Although the host response was variable between mice, it was significantly more similar between mice which were transplanted with the same donor hepatocytes, animals C585 and C572 (Figure 2A). In addition, individual samples from the same liver showed remarkably similar induction of ISGs, in both the identity of ISGs and level of induction.

Interestingly, there appeared to be an inverse association between the total number of differentially regulated genes and the number of up-regulated IFN genes. Animals with high numbers of differentially expressed genes (C585 and C572) tended to have lower numbers of induced IFN-regulated genes, while animals with low numbers of differentially expressed genes (B473 and D196) tended to have high numbers of induced IFN-regulated genes (Figure 3). It is possible that induction of an effective innate antiviral response during the acute phase of HCV infection can limit the spread of infection throughout the liver and/or the level of HCV replication in already infected hepatocytes. This may correlate with a less pronounced effect on the host cell as measured by fewer changes in intrahepatic gene expression. In support of this, quantitation of the intrahepatic HCV viral loads indicated that a strong IFN response was generally associated with lower levels of HCV RNA, whereas a weak IFN response was generally associated with higher levels of HCV RNA (Figure 3). The main exception to this was animal D196, which had a strong IFN response and relatively high levels of intrahepatic HCV RNA. This animal also differed from the remaining animal in that it was infected for a shorter period of time (4 wk).

**Figure 3:**
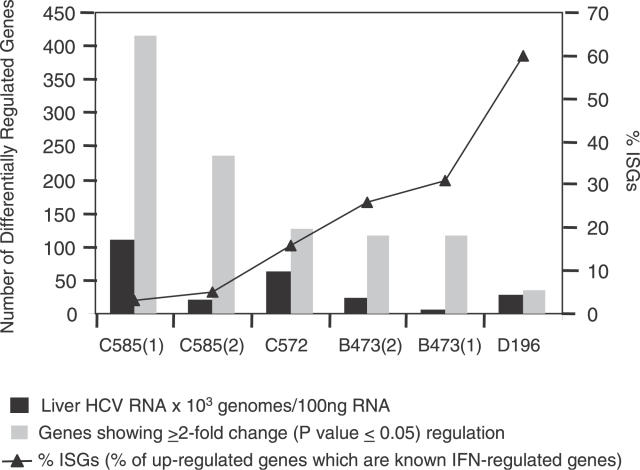
Association between IFN Response, HCV RNA Levels, and Effect on Host Gene Expression in HCV-Infected Mice Intrahepatic HCV RNA levels were determined by quantitative RT-PCR as described in Materials and Methods. Total number of genes showing increased expression in HCV-infected mice relative to donor-matched uninfected mice is shown in gray bars, while the percent of induced genes that are IFN regulated is shown using black triangles.

### HCV Infection Affects Multiple Aspects of Lipid Metabolism in a Host-Specific Manner

HCV-infected mice showed an induction in the expression of many lipid metabolism genes. Similar to what is observed in HCV-infected chimpanzees [9], this modulation appeared to be host specific and tended to correlate with liver HCV RNA levels. Two HCV-infected animals, B473 and D196, only showed significant up-regulation of PLA2G2A, an acute phase protein which is associated with propagation of inflammation. In contrast, both C585 and C572, which contain the same donor hepatocytes and, in general, had higher levels of intrahepatic HCV RNA, showed an induction of a number of genes encoding proteins/enzymes associated with many aspects of lipid metabolism. Specifically, there was increased expression of enzymes involved in fatty acid biosynthesis as well as proteins involved in the transport of fatty acids, suggesting that HCV infection was inducing fatty acid biosynthesis (Figure 4A). Animals C585 and C572 exhibited an up-regulation in the expression of many enzymes responsible for cholesterol synthesis (FDPA, HMG-CS1, HMG-CS2, HMG-CoA reductase), including some that catalyze the rate-limiting steps in the synthesis pathway, such as lanosterol synthetase (LSS) and squalene epoxidase (SQLE) (Figure 4A). There was also an increase in the expression of proteins involved in cholesterol transport and catabolism, SORL1 and CYP7A1, suggesting that the hepatocytes are trying to deal with the excess cholesterol by removing it from the cell.

**Figure 4:**
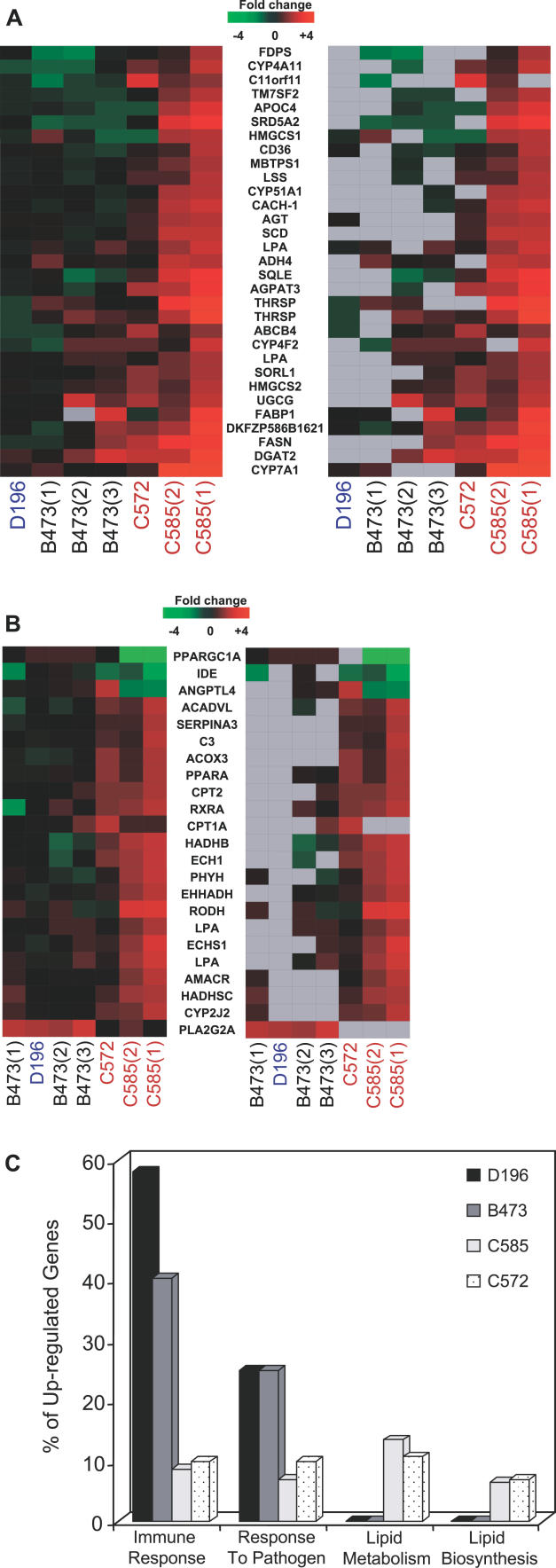
Expression of Genes Associated with Lipid Metabolism in HCV-Infected Mice Expression of genes associated with fatty acid and cholesterol biosynthesis (A) and peroxisomes, inflammation, and β-oxidation (B) that are at least 2-fold regulated with *p* ≤ 0.05 in at least one of seven experiments. See legend of Figure 1 for details on clustering, the applied color scheme for the fold changes in the mRNA levels, and experiment representation. (C) Association between host defense response and lipid biosynthesis. Genes were selected as differentially expressed based on at least a 2-fold change, *p* ≤ 0.05, and placed into functional categories using Fatigo software. Graph depicts percent of differentially regulated genes associated with each category for each experiment. Results from four independent (individual animals) experiments comparing donor-matched HCV-infected and naïve tissue are shown.

Interestingly, there was increased expression of genes associated with peroxisomes, which contain enzymes for respiration and cholesterol/lipid metabolism (Figure 4B), specifically in animals in which both fatty acid and cholesterol biosynthesis appeared to be increased. These include peroxisome proliferator-activated receptor alpha (PPARα) as well as genes known to be regulated by PPARα, such as ANGPTL4, ECH1, PHYH, LBP, and ACOX3. In addition, there was increased expression of enzymes which mediate β-oxidation, the process of breaking down fatty acids to generate energy. Animal C585 in particular had increased expression of almost all the main enzymes involved in mitochondrial β-oxidation (Figure 4B).

The reason for the host-specific nature of the regulation of lipid metabolism genes is unclear. One possibility is that certain donor hepatocytes had a higher baseline expression of lipid biosynthesis genes, allowing for higher levels of HCV replication during the initial stage of infection. However, quantitative RT-PCR analysis did not reveal significant differences in the expression of ten lipid biosynthesis genes among the uninfected animals (unpublished data). Alternatively, it may be related to the nature of the IFN response. There was an inverse association between the induction of the host defense response and induction of fatty acid and cholesterol biosynthesis genes in HCV-infected animals (Figure 4C). Animals B473 and D196 both had strong IFN responses and no induction of genes associated with fatty acid or cholesterol biosynthesis. In contrast, animals C585 and C572 had a less intense IFN response but exhibited induction of lipid biosynthesis genes. Although animals C585 and C572 contain the same donor hepatocytes and exhibited very similar host responses to HCV infection, C572 appeared to have a slightly stronger induction of the IFN response (Figure 2A). Interestingly, C572 had a corresponding lower level of intrahepatic HCV RNA and less induction of lipid metabolism genes.

### Potential Link between Disturbances in Lipid Metabolism and Oxidative Stress

The gene expression data suggest that, in at least two of the animals, there was an increase in both the synthesis and β-oxidation of fatty acids. Lipid peroxidation produces reactive oxidative species (ROS), such as H_2_O_2_, which can cause damage to cellular proteins. In support of this, there is evidence for oxidative stress specifically in animals which showed an induction of the expression of enzymes in the β-oxidation pathway (Figure 5). The level of induction of these stress-response genes correlated with the expression of the β-oxidation enzymes. Animal C585, which showed the greatest induction, also showed the greatest induction of genes associated with oxidative stress (Figure 5, pink text). In contrast, the other samples showed either less effect (C572) or no effect (B473 and D196) (Figure 5). Some of the genes listed in Figure 5 are induced in response to DNA damage (shown in blue text), possibly caused by ROS. Again, the induction of oxidative stress appeared to correlate with a weak IFN response.

**Figure 5:**
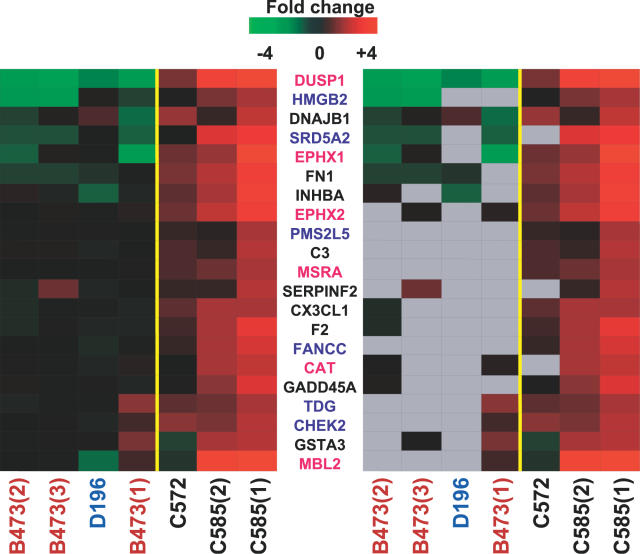
Association between Fatty Acid β-Oxidation and Expression of Genes Implicated in the Stress Response Expression of genes associated with stress response. See legend of Figure 1 for details on clustering, the applied color scheme for the fold changes in the mRNA levels, and experiment representation. Genes implicated in oxidative stress are shown in pink text, while genes induced in response to DNA damage are shown in blue text.

### Comparison of Host Response to Infection in HCV-Infected Mice and Patients

One limitation of gene expression studies using biopsies from HCV-infected individuals is the inability to ascertain whether certain gene expression changes are the direct result of viral replication or are the result of HCV-specific adaptive immune responses. Because the mice used in this study are incapable of generating an HCV-specific adaptive immune response, comparison of HCV-induced gene expression changes in these animals with gene expression changes in HCV-infected patients provides a way of distinguishing viral and immune-mediated effects. To facilitate a more direct comparison, microarray experiments were performed where HCV-infected liver tissue from patients (*n* = 5) and animals (*n* = 5), as well as uninfected animals (*n* = 3), was independently compared to a common reference. The reference used was a pool of RNA from eight normal uninfected liver samples [3,5,17]. Morphological and histological examinations of mouse tissue were also performed and the results compared to results obtained from patient samples. Animals used for histological analysis were independent of those used for the gene expression studies.

When the gene expression profiles of HCV-infected patients and mice were compared, the most notable difference was seen in the expression of immune cell–specific genes. The mouse samples showed extensive down-regulation of genes whose expression is mainly restricted to immune cells, including T and B lymphocytes, natural killer cells, and neutrophils (Figure 6). This down-regulation was apparent in both HCV-infected and uninfected animals. Normal liver has a small population of resident lymphocytes and it is possible that a small percentage of this population is present in the hepatocyte suspension that is transplanted into the animals. However, the strong, uniform down-regulation of lymphocyte-specific genes suggests that if this occurs, the lymphocytes are not sustained long in the microenvironment of the mouse liver, resulting in an underrepresentation of the genes in the mouse liver tissue compared to normal liver. In contrast, the patient samples actually showed an up-regulation of some of these genes, likely due to the increased infiltration of lymphocytes that occurs during chronic HCV infection [5, 17] (Figure 6).

**Figure 6:**
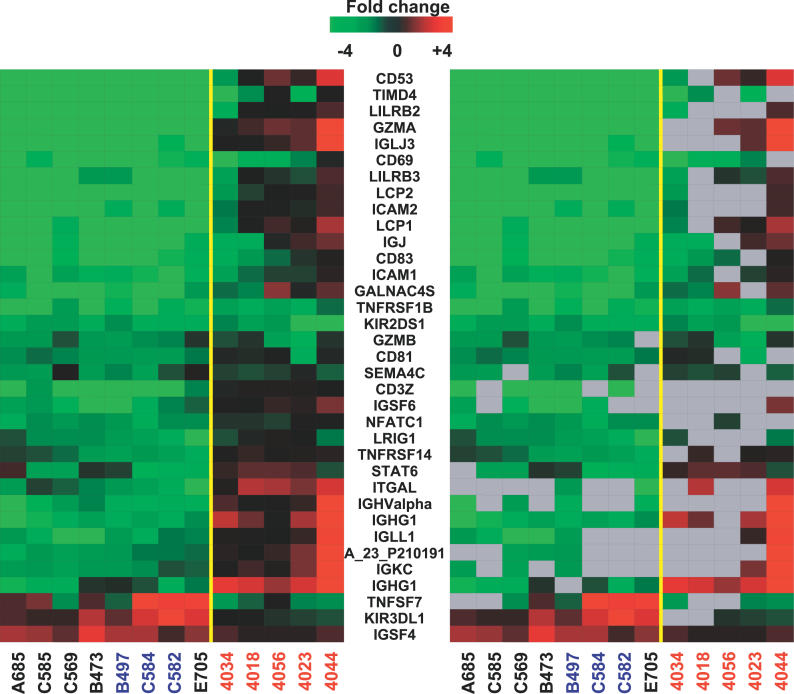
Expression of Markers Associated with Immune Cells in HCV-Infected Mice and Patients Expression of genes that were at least 2-fold regulated with *p* ≤ 0.05 in at least three of 13 experiments comparing uninfected mouse (blue text) and HCV-infected liver tissue (mouse samples indicated by black text, patient samples indicated with red text) to a normal liver reference. See legend of Figure 1 for details on clustering, the applied color scheme for the fold changes in the mRNA levels, and experiment representation.

The expressions of genes from the IFN-signaling pathway were strikingly similar between HCV-infected mice and patients (Figure 7A and 7B, respectively), indicating that HCV is triggering the same host innate antiviral response pathways in both populations. Both showed an induction of genes associated with creating an intracellular antiviral state, such as those involved in antigen processing and presentation on MHC class I molecules. They also showed similar induction of ISGF3, IRF3, IRF1, and nuclear factor-κB target genes. This suggests that despite the absence of an adaptive immune response, this model is well suited for investigating the interplay between HCV and the innate antiviral response. Again, the only significant differences between HCV-infected animals and patients are seen in the expression of genes specifically associated with immune cells, such as CD74 and MHC class II, which is likely due to the absence of these cell types in the mouse.

**Figure 7:**
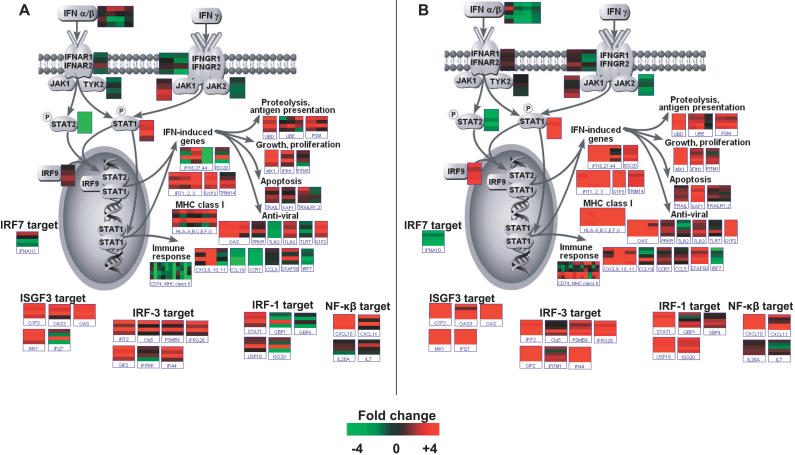
Induction of IFN-Signaling Pathways in HCV-Infected Mice and Patients Pathway Builder (Protein Lounge) and the Pathway feature of Resolver were used to visualize the expression of genes from the IFN signaling pathway in HCV-infected mice (A) and patients (B). The expression profiles of individual genes are shown in boxes, and each bar within the box represents one experiment. Color schemes are as indicated in Figure 1. The reference for each microarray experiment was a pool of normal, uninfected human liver tissue.

Liver sections from HCV-infected (*n* = 10) and uninfected (*n* = 4) animals were subjected to histopathological analysis and the results compared to clinical HCV specimens (*n* = 5). Immunohistochemical assays were performed to detect FAS and activated Caspase-3 expression, and the results were scored in a semiquantitative manner. Similar to what was seen in liver biopsies from HCV-infected patients (Figure 8A), FAS expression was increased in response to HCV infection as shown by the strong membrane-associated staining using an anti-FAS antibody (Figure 8B). In contrast, no staining was observed in either uninfected animals (Figure 8C) or mouse liver cells, indicating that FAS expression is specifically induced by HCV infection and that the antibody is specific to human FAS (unpublished data). Because FAS-associated apoptosis, mediated by FAS-L expressing cytotoxic T lymphocytes, is thought to play a key role in HCV-associated liver disease [18–20], the level of apoptosis was examined using both Caspase-3 activation immunohistochemistry and lobular apoptotic body count. Surprisingly, there was a trend toward increasing signals of apoptosis in HCV-infected mice (apoptotic bodies are indicated by arrows in Figure 8D) relative to donor-matched uninfected animals. There also appeared to be increased inflammatory activity (indicated by arrowheads in Figure 8D) in the hepatic lobules as well as hepatocyte swelling, indicative of hepatocellular injury, in the HCV-infected mice. The inflammatory cells consisted predominantly of mononuclear and Kupffer cells. These findings are similar to what is observed in early phase hepatitis C infection, as well as in early recurrent hepatitis C after liver transplantation [21–23]. Not surprisingly, Masson trichome stain did not show any significant portal fibrosis in either HCV-infected or uninfected mice (data not shown). Steatosis was often observed specifically in the human hepatocytes of the chimeric mice (seen in Figure 8) but was apparent in both naïve and HCV-infected animals, indicating that it is specific to the donor hepatocytes but not necessarily HCV infection.

**Figure 8:**
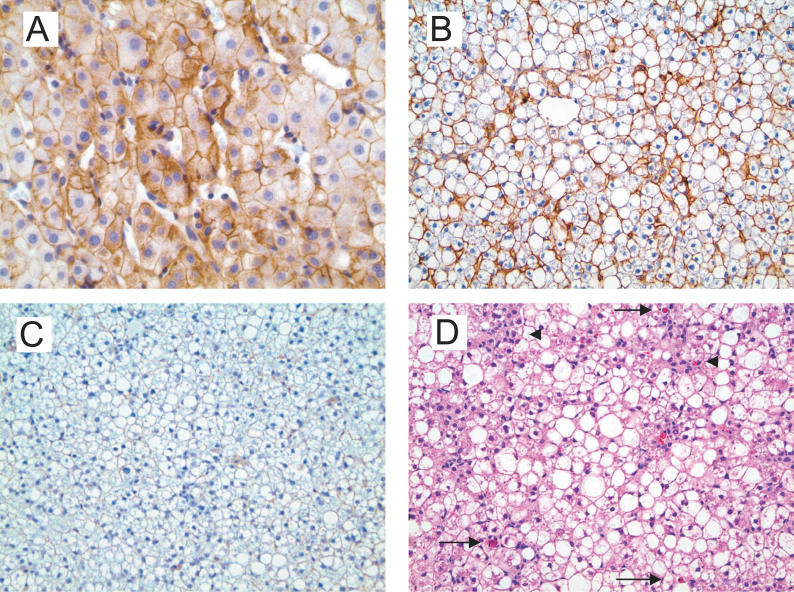
Morphological and Immunohistochemical Analysis of HCV-Infected Mice (A) Immunohistochemical staining for FAS in liver tissue from an HCV-infected patient showing diffuse strong membranous staining. Magnification ×200. (B) Immunohistochemical staining for FAS in liver tissue from an HCV-infected mouse. Magnification ×40. (C) Immunohistochemical staining for FAS in liver tissue from an uninfected mouse. Magnification ×200. (D) Hematoxylin and eosin staining of liver tissue from an HCV-infected mouse. The apoptotic bodies are indicated by arrows, and the inflammatory foci are indicated by arrowheads. Magnification ×200.

## Discussion

This study represents the first report of transcriptional profiling of HCV-induced gene expression changes in the chimeric SCID-beige/Alb-uPA mouse model. It is unique in that it examines the host response to a single HCV inoculum in multiple donor hepatocytes and demonstrates for the first time that host factors play a key role in this response. Mice containing hepatocytes from different donors exhibited very different transcriptional changes despite being infected with the same HCV inoculum. Animals containing the hepatocytes from the same donor showed a much more similar response and individual samples from the same liver were remarkably similar. Together, these results suggest that host factors are influencing the response to HCV infection.

Whereas all HCV-infected animals showed activation of the host innate antiviral signaling pathways, as indicated by the induction of ISGs, this response was highly variable between mice containing hepatocytes from different donors. The differences in the level of activation of ISGs appeared to be independent of viral factors including serum HCV titers and duration of infection. This variation in the host innate antiviral response is consistent with previous gene expression studies of liver biopsies from HCV-infected patients [5,17]. However, those studies were complicated by variation in both virus inoculum and length of infection, making it difficult to determine the individual roles of viral and host factors in this host response variation. Currently, much emphasis is placed on the role of viral factors in regulating the host innate response to infection, and there is evidence that HCV is able to attenuate IFN signaling by multiple mechanisms (reviewed in [24]). These mechanisms include HCV NS3/4A-mediated antagonism of IRF3 activation through cleavage of adaptor proteins of the RIG-I and TLR3 double-strand RNA signaling pathways, both of which function to attenuate ISG expression [25–29]. In addition, the HCV NS5A and E2 have been shown to bind to and inhibit the catalytic activity of PKR, effectively disrupting PKR-dependent translational control and signaling actions [30–32]. The differences in the number and intensity of induction of ISGs between animals containing hepatocytes from different donors argue that host factors can also influence the effectiveness of the innate immune response.

The induction of ISGs in the mouse hepatocytes of HCV-infected mice was somewhat surprising. Although the mouse hepatocytes are not susceptible to HCV infection, the close proximity of human and mouse hepatocytes [11] makes it is feasible that this response in mouse hepatocytes is due to cytokines released from neighboring HCV-infected human cells.

The ability of HCV to maintain a persistent infection despite activation of the innate antiviral response is intriguing. While transcriptional profiling indicates that there was an IFN response in the livers of HCV-infected animals, it does not provide information about where the response is coming from. It is possible that the induction of ISGs measured on the microarrays is originating primarily from uninfected human cells, limiting the spread of HCV throughout the liver. This would explain why mice which had strong IFN responses tended to have lower levels of intrahepatic HCV RNA and vice versa.

In general, the effect of HCV replication on host gene expression in the mice was not that extreme, ranging from 60 to 653 differentially expressed genes in individual animals, and was similar to what is observed in HCV-infected chimpanzees [9]. This is perhaps not surprising, as it is unlikely that HCV could establish a chronic infection characterized by slowly progressive liver disease if infection of hepatocytes severely affected the normal functions of the cell. Interestingly, disturbances in the expression of lipid metabolism genes were observed in two HCV-infected animals transplanted with the same donor hepatocytes. Lipid disturbances have been well documented in chronically infected individuals, including hypocholesteremia and steatosis (accumulation of fat droplets in hepatocytes), suggesting that modulation of cellular lipid metabolism plays an important role in the HCV life cycle [33–36]. HCV replication during acute infection of chimpanzees is associated with a host-specific regulation of lipid metabolism genes, including increased expression of genes required for fatty acid and cholesterol synthesis [6, 9]. Inhibition of cholesterol synthesis by treatment with inhibitors of HMG-CoA reductase decreases HCV replication in the replicon system, indicating that the cholesterol biosynthetic pathway regulates HCV replication [9, 37]. Cholesterol is a vital component of cellular membranes, and it is feasible that HCV induces cholesterol biosynthesis to accommodate both progeny virion formation and the production of intracellular membranes which are the site of HCV replication. In addition, a geranylgeranylated host protein, FBL2, was recently shown to form a complex with NS5A which is required for HCV replication [38]. Induction of the cholesterol biosynthetic pathway would therefore serve to increase biosynthesis of cholesterol and isoprenoids (geranylgeranyl groups), both of which play important roles in HCV replication. This might explain why the greatest induction of these genes is seen in animals which generally have higher levels of intrahepatic HCV RNA.

The mechanism by which HCV regulates the expression of lipid metabolism genes is unknown. Many of the genes are regulated by the transcription factor sterol regulatory element binding protein (SREBP), which binds to an SRE element present in the promoters of target genes [39,40]. SREBP is normally localized to the endoplasmic reticulum membrane and is activated by two cleavage events which release it from the plasma membrane, allowing it to enter the nucleus. This cleavage activation is regulated by sterol levels through the action of SCAP, which regulates the activity of the S1P, the protease which catalyzes the first cleavage event [40–42]. While the expression of SREBP was not significantly increased, the expression of genes encoding two proteins responsible for regulating its activation, SCAP and S1P, was increased specifically in the two animals which showed an increase in SRE-inducible genes. The expression of SCAP was increased 1.5-fold (*p* < 0.001), while SIP was increased greater than 2-fold (*p* < 0.001). Increased expression of SCAP and S1P may coincide with increased cleavage and release of SREBP from the endoplasmic reticulum membrane, allowing it to travel to the nucleus and promote transcription of SRE-regulated genes.

Increased expression of lipid biosynthesis genes in HCV-infected mice was associated with increased expression of both β-oxidation enzymes and oxidative stress-inducible genes, suggesting that lipid peroxidation was generating higher levels of ROS in certain animals. The production of excessive ROS potentially impacts both the development of fibrosis and HCC. Indeed, a preliminary study has demonstrated changes in levels of proteins associated with lipid metabolism and oxidative stress specifically in a liver biopsy sample from a patient who developed fibrosis following liver transplantation [43]. These changes were not observed in biopsy samples from patients who did not develop fibrosis. It is possible that the apoptosis of hepatocytes observed in HCV-infected mice is linked to the oxidative stress that results from increased lipid peroxidation. Indeed, enhanced production of ROS can induce cell death through the activation of transcription factors such as nuclear factor-κB [44]. In addition, oxidative stress can initiate fibrogenesis by increasing procollagen type I gene expression [44].

Evidence of both oxidative stress and apoptosis indicates that the liver pathology associated with chronic HCV infection may not be caused exclusively by an HCV-specific cell-mediated immune response. The fact that this effect tended to be inversely related to the strength of the innate antiviral response and corresponding HCV RNA level is intriguing, although additional studies with a larger cohort of animals is needed to verify this association. To date, there has been no clear association between HCV level and severity of liver disease [45,46]. However, there is circumstantial evidence that suggests higher HCV levels may correlate with enhanced fibrosis. Immune suppression, including that caused by immunosuppressive drugs and HIV coinfection, is often associated with a more accelerated progression of liver disease [47–52]. This is somewhat perplexing, given that the pathology of HCV infection is thought to be primarily immune mediated [2]. Both HIV coinfected individuals and those on immunosuppressive drugs following liver transplant tend to have higher serum levels of HCV RNA, presumably due to the inability of the immune system to control HCV replication [53]. Increased levels of intrahepatic HCV replication have been shown to correlate with liver disease severity [54]. In addition, a recent report describing transcriptional profiling of serial biopsy samples from HCV-infected patients following liver transplant demonstrated a correlation between the host IFN response and development of fibrosis [5]. Similar to the findings in HCV-infected mice, an impaired IFN response and accumulation of high numbers of differentially expressed genes are observed specifically in patients with rapidly progressing fibrosis. These results suggest that the nature of the innate antiviral response during the acute phase of infection not only plays a role in determining the outcome of infection but also is an important contributing factor in the progression of liver disease. Induction of a strong IFN response during the acute phase of HCV infection may limit the spread of infection throughout the liver and/or the level of HCV replication in already infected hepatocytes to a level which does not significantly impact hepatocyte function. This argues that suppression of HCV replication, even in the absence of complete viral clearance, could improve clinical outcome. Comparison of gene expression data from HCV-infected mice and the transplant patients also suggests that despite the lack of fibrosis in HCV-infected mice, it is a valuable model in which to investigate the role of host factors in the development of liver disease. In particular, the model can be used to study aspects of the innate antiviral immune response that potentially limit HCV replication to minimize its effect on cellular functions.

## Materials and Methods

### Dissection of mouse livers and isolation of RNA and genomic DNA.

All mice were treated according to Canadian Council on Animal Care guidelines. SCID-beige/Alb-uPA mice were transplanted with human primary hepatocytes and then infected with HCV as described previously [10,12]. Mice were killed by cervical dislocation, and the livers were excised, dissected into small pieces, and then snap frozen in N_2_. Total RNA was then isolated according to a standard TRIZOL procedure. Genomic DNA was isolated from the interphase and phenol/chloroform phase according to the manufacturer's specifications (Invitrogen, Carlsbad, California, United States). Both RNA and genomic DNA were isolated from the same samples.

### PCR of human and mouse genomic DNA.

Genomic DNA from the liver of a SCID-beige/Alb-uPA mouse containing human hepatocytes was amplified by PCR with primers specific for the fourth and fifth exons of the gene for the succinyl-CoA synthetase alpha subunit (SCS-α). The PCR conditions were 0.01% (w/v) gelatin, 50 mM KCl, 1.5 mM MgCl_2_, 10 mM Tris (pH 8.3), 0.2 mM concentration each of dATP, dGTP, dCTP, and dTTP, 2.5 U of Taq polymerase, and 0.25 μM each of 5′-TTGTGAATATGGCCAGGCATG (SCS-ex5) and 5′-CAGGAGCAACGGCTTCTGTC (SCS-ex4). The underlined nucleotide denotes the position of the nucleotide that is present in the human sequence but not in the mouse sequence. The PCR cycles were 5 min at 95 °C, followed by 30 cycles of (30 s at 95 °C, 30 s at 45 °C, and 30 s at 72 °C). The resulting PCR products were separated by electrophoresis in a 1.5% agarose gel, excised, purified using a QIAquick gel extraction kit (Qiagen, Valencia, California, United States), cloned into pCR4 TOPO according to the manufacturer's specifications (Invitrogen), and sequenced. The cloned fragments from mouse and human were 452 bp and 384 bp, respectively. For measurement of the relative amount of human and mouse SCS-α, the PCR conditions were as follows: 1.2 μg of genomic DNA, 20 mM Tris (pH 8.4), 50 mM KCl, 2 mM MgCl_2_, 0.2 mM concentration each of dATP, dGTP, dCTP, and dTTP, 2.5 U of Taq polymerase, and 0.25 μM concentration each of SCS-ex4 and SCS-ex5. PCR cycles were 10 min at 95 °C followed by 40 cycles of (45 s at 95 °C, 45 s at 68 °C, and 45 s at 72 °C) and a final extension of 10 min at 72 °C. The cloned plasmids were used as standards and were varied from 3.5 pg human/28 pg mouse to 28 pg human/3.5 pg mouse per reaction. To determine the relative amount of human SCS-α gene in the sample, the PCR products were separated by electrophoresis using a 2.5% agarose gel and then stained with ethidium bromide. The bands were quantified, a correction was made for the size difference, and the results were plotted on a standard curve generated using the plasmid controls.

### Expression microarray format and data analysis.

Microarray format, protocols for probe labeling, and array hybridization are described at http://expression.microslu.washington.edu. Briefly, a single experiment comparing two mRNA samples was done with four replicate human 1A (V2) 22K oligonucleotide expression arrays (Agilent Technologies, Palo Alto, California, United States) using the dye label reverse technique. This allows for the calculation of mean ratios between expression levels of each gene in the analyzed sample pair, standard deviation, and *p*-values for each experiment. Spot quantitation, normalization, and application of a platform-specific error model were performed using Agilent's Feature Extractor software, and all data were then entered into a custom-designed database, Expression Array Manager, and then uploaded into Rosetta Resolver System 4.0.1.0.10 (Rosetta Biosoftware, Kirkland, Washington, United States) and Spotfire Decision Suite 7.1.1 (Spotfire, Somerville, Massachusetts, United States). Data normalization and the Resolver Error Model are described on the Web site (http://expression.microslu.washington.edu). This Web site is also used to publish all primary data in accordance with the proposed MIAME standards [55]. Selection of genes for data analysis was based on a greater than 95% probability of being differentially expressed (*p* ≤ 0.05) and a fold change of 2 or greater. The resultant false-positive discovery rate was estimated to be less than 0.1% (KAW, unpublished data).

### Immunohistochemistry.

Hematoxylin and eosin staining was performed according to the standard procedures. The inflammatory activity and degree of fibrosis in the liver were graded and staged, respectively, according to a modified Batts and Ludwig scoring system [56]. In particular, the inflammatory activity was scored in the portal tracts and hepatic lobules, respectively. The degree of fatty change was scored as 0, <5%; 1, 6% to 33%; 2, 34% to 66%; and 3, >66%. Average lobular apoptotic bodies were obtained by counting the apoptotic bodies in five different fields at ×10 objective. Immunohistochemical analyses were performed on paraffin sections from formalin-fixed material using monoclonal antibodies against FAS (1:80 dilution; Novocastro, Newcastle, United Kingdom) and activated caspase (1:200 dilution; Cell Signaling Technology, Beverly, Massachusetts, United States). Following blockage of endogenous peroxidase activities, dewaxed and rehydrated slides were subjected to epitope retrieval performed in 0.01 M citrate (pH 6.0) in a commercial microwave oven for 15 min. All stains were developed using a standard avidin-biotin-peroxidase detection system (Vectastain Elite ABC kit; Vector Laboratories, Burlingame, California, United States) with 3,3′-daminobenzidine-4 HCl as chromogen. Immunohistochemical studies for FAS and activated Caspase-3 were assessed in a semiquantitative fashion. Briefly, the intensity of FAS and activated Caspase-3 staining was graded as 0, none; 1, 1% to 25%; 2, 26% to 50%; 3, 51% to 75%; and 4, 76% to 100%. Masson trichome stain was performed according to routine procedures.

### Quantitative RT-PCR.

Quantitative RT-PCR was used to validate the gene expression changes and measure intrahepatic HCV RNA. Total RNA samples were treated with DNA-free DNase Treatment and Removal Reagents (Ambion, Austin, Texas, United States). Reverse transcription was performed using random hexamer primers and Taqman RT reagents (Applied Biosystems, Foster City, California, United States). RT-PCR was performed using an ABI 7500 Real Time PCR system and Taqman chemistry. Each target was run in quadruplicate with Taqman 2X PCR Universal Master Mix and a 20-μl total reaction volume. Primer and probe sets for relative quantification were selected from the Assays-on-Demand product list (Applied Biosystems) including two endogenous controls: GAPDH and 18S ribosomal RNA. Quantification of each gene, relative to the calibrator, was calculated by the instrument, using the equation 2^−ΔCT^ within the Applied Biosystems Sequence Detections Software version 1.3. Probes used for analysis (Applied Biosystems) were human genes: eukaryotic 18S rRNA (catalog No. Hs99999901_s1), IFIT1 (catalog No. Hs00356631_g1), ITITM1 (catalog No. Hs00705137_s1), MX1 (catalog No. Hs00182073_m1), OAS2 (catalog No. Hs00159719_m1), OASL (catalog No. Hs00388714_m1), and STAT1 (catalog No. Hs00234829_m1); and mouse genes: IFIT1 (catalog No. Mm00515153_m1), IFIT1 (catalog No. Mm00515153_m1), IFITM1 (catalog No. Mm00850040_g1), MX1 (catalog No. Mm00487796_m1), OAS2 (catalog No. Mm00460961_m1), OASL (catalog No. Mm00496187_m1), and STAT1 (catalog No. Mm00439518_m1).

Primer and probe sets for absolute quantification of intrahepatic viral load were designed based on sequences of HCV 1a armored RNA (Ambion Diagnostics, Austin, Texas, United States) using Primer Express (version 3). A standard curve was made from six serial dilutions of HCV 1a armored RNA (Ambion Diagnostics) with a known viral copy number. The PCR efficiency was determined by the slope of the standard curve Standard curve analysis and viral load weres determined using the Applied Biosystems SDS Software 1.3 (Applied Biosystems). Total RNA was DNase treated prior to cDNA synthesis via reverse transcription, and all samples were processed with equal mass amounts of total RNA [57]. All measurements were taken in quadruplicate with negative and nontemplate controls. Primer and probe sets consisted of forward: CAC TCC CCT GTG AGG AAC TAC TG; reverse: GCT GCA CGA CAC TCA TAC TAA CG; and probe: 6FAM-TTC ACG CAG AAA GC-MGBNFQ, and were designed from the 5′ UTR using Primer Express 3.0 (Applied Biosystems). Quantification of HCV RNA levels was performed on the same total RNA sample that was used for the microarray experiments.

## Abbreviations

Alb: albumin
HCC: hepatocellular carcinoma
HCV: hepatitis C virus
IFN: interferon
ISG: interferon-stimulated gene
MHC: major histocompatibility complex
NK: natural killer
ROS: reactive oxygen species
RT: real-time
SCID: severe combined immunodeficiency disorder
SREBP: sterol regulatory element binding protein
uPA: urokinase plasminogen activator

## Note Added in Proof

Reference 58 is cited out of order in the article because it was added while the article was in proof.

## References

[ppat-0020059-b001] Alter MJH, Margolis HS, Krawczynski K, Judson F, Mares A (1992). The natural history of community-acquired hepatitis C in the United States. N Engl J Med.

[ppat-0020059-b002] Nelson DR (2001). The immunopathogenesis of hepatitis C virus infection. Clin Liver Dis.

[ppat-0020059-b003] Smith MW, Yue ZN, Geiss GK, Sadovnikova NY, Carter VS (2003). Identification of novel tumor markers in hepatitis C virus-associated hepatocellular carcinoma. Cancer Res.

[ppat-0020059-b004] Smith MW, Yue ZN, Korth MJ, Do HA, Boix L (2003). Hepatitis C virus and liver disease: Global transcriptional profiling and identification of potential markers. Hepatology.

[ppat-0020059-b005] Smith MW, Walters KA, Korth MJ, Fitzgibbon M, Proll S (2006). Gene expression patterns that correlate with hepatitis C and early progression to fibrosis in liver transplant recipients. Gastroenterology.

[ppat-0020059-b006] Bigger CB, Brasky KM, Lanford RE (2001). DNA microarray analysis of chimpanzee liver during acute resolving hepatitis C virus infection. J Virol.

[ppat-0020059-b007] Bigger CB, Guerra B, Brasky KM, Hubbard G, Beard MR (2004). Intrahepatic gene expression during chronic hepatitis C virus infection in chimpanzees. J Virol.

[ppat-0020059-b008] Honda M, Kaneko S, Kawai H, Shirota Y, Kobayashi K (2001). Differential gene expression between chronic hepatitis B and C hepatic lesion. Gastroenterology.

[ppat-0020059-b009] Su AI, Pezacki JP, Wodicka L, Brideau AD, Supekova L (2002). Genomic analysis of the host response to hepatitis C virus infection. Proc Natl Acad Sci U S A.

[ppat-0020059-b010] Mercer DF, Schiller DE, Elliott JF, Douglas DN, Hao C (2001). Hepatitis C virus replication in mice with chimeric human livers. Nat Med.

[ppat-0020059-b011] Meuleman P, Libbrecht L, De Vos R, de Hemptinne B, Gevaert K (2005). Morphological and biochemical characterization of a human liver in a uPA-SCID mouse chimera. Hepatology.

[ppat-0020059-b012] Hsu EC, Hsi B, Hirota-Tsuchihara M, Ruland J, Iorio C (2003). Modified apoptotic molecule (BID) reduces hepatitis C virus infection in mice with chimeric human livers. Nat Biotechnol.

[ppat-0020059-b013] Lindenbach BD, Meuleman P, Ploss A, Vanwolleghem T, Syder AJ (2006). Cell culture-grown hepatitis C virus is infectious in vivo and can be recultured in vitro. Proc Natl Acad Sci U S A.

[ppat-0020059-b014] Wiese M, Grungreiff K, Guthoff W, Lafrenz M, Oesen U (2005). Outcome in a hepatitis C (genotype 1b) single source outbreak in Germany—A 25-year multicenter study. J Hepatol.

[ppat-0020059-b015] Mas A, Ulloa E, Bruguera M, Furcic I, Garriga D (2004). Hepatitis C virus population analysis of a single-source nosocomial outbreak reveals an inverse correlation between viral load and quasispecies complexity. J Gen Virol.

[ppat-0020059-b016] Helbig KJ, Lau DT, Semendric L, Harley HA, Beard MR (2005). Analysis of ISG expression in chronic hepatitis C identifies viperin as a potential antiviral effector. Hepatology.

[ppat-0020059-b017] Walters KA, Smith MW, Pal S, Thompson JC, Thomas MJ (2006). Identification of a specific gene expression pattern associated with HCV-induced pathogenesis in HCV- and HCV/HIV-infected individuals. Virology.

[ppat-0020059-b018] Hayashi N, Mita E (1999). Involvement of Fas system-mediated apoptosis in pathogenesis of viral hepatitis. J Viral Hepatol.

[ppat-0020059-b019] Hiramatsu N, Hayashi N, Katayama K, Mochizuki K, Kawanishi Y (1994). Immunohistochemical detection of Fas antigen in liver tissue of patients with chronic hepatitis C. Hepatology.

[ppat-0020059-b020] Pianko S, Patella S, Ostapowicz G, Desmond P, Sievert W (2001). Fas-mediated hepatocyte apoptosis is increased by hepatitis C virus infection and alcohol consumption, and may be associated with hepatic fibrosis: Mechanisms of liver cell injury in chronic hepatitis C virus infection. J Viral Hepat.

[ppat-0020059-b021] Gerber MA (1994). Pathology of hepatitis C. FEMS Microbiol Rev.

[ppat-0020059-b022] Goodman ZD, Ishak KG (1995). Histopathology of hepatitis C virus infection. Semin Liver Dis.

[ppat-0020059-b023] Charlton M, Wiesner R (2004). Natural history and management of hepatitis C infection after liver transplantation. Semin Liver Dis.

[ppat-0020059-b024] Gale M, Foy EM (2005). Evasion of intracellular host defence by hepatitis C virus. Nature.

[ppat-0020059-b025] Foy E, Li K, Wang C, Sumpter RJ, Ikeda M (2003). Regulation of interferon regulatory factor-3 by the hepatitis C virus serine protease. Science.

[ppat-0020059-b026] Foy E, Li K, Sumpter R, Loo YM, Johnson CL (2005). Control of antiviral defenses through hepatitis C virus disruption of retinoic acid-inducible gene-I signaling. Proc Natl Acad Sci U S A.

[ppat-0020059-b027] Li K, Foy E, Ferreon JC, Nakamura M, Ferreon AC (2005). Immune evasion by hepatitis C virus NS3/4A protease-mediated cleavage of the Toll-like receptor 3 adaptor protein TRIF. Proc Natl Acad Sci U S A.

[ppat-0020059-b028] Sumpter R, Loo YM, Foy E, Li K, Yoneyama M (2005). Regulating intracellular antiviral defense and permissiveness to hepatitis C virus RNA replication through a cellular RNA helicase, RIG-I. J Virol.

[ppat-0020059-b029] Ferreon JC, Ferreon AC, Li K, Lemon SM (2005). Molecular determinants of TRIF proteolysis mediated by the hepatitis C virus NS3/4A protease. J Biol Chem.

[ppat-0020059-b030] Taylor DR, Shi ST, Romano PR, Barber GN, Lai MMC (1999). Inhibition of the interferon-inducible protein kinase PKR by HCV E2 protein. Science.

[ppat-0020059-b031] Noguchi T, Satoh S, Noshi T, Hatada E, Fukuda R (2002). Effects of mutation in hepatitis C virus nonstructural protein 5A on interferon resistance mediated by inhibition of PKR kinase activity in mammalian cells. Microbiol Immunol.

[ppat-0020059-b032] Gale M, Blakely CM, Kwieciszewski B, Tan SL, Dossett M (1998). Control of PKR protein kinase by hepatitis C virus nonstructural 5A protein: Molecular mechanisms of kinase regulation. Mol Cell Biol.

[ppat-0020059-b033] Siagris D, Christofidou M, Theocharis GJ, Pagoni N, Papadimitriou C (2006). Serum lipid pattern in chronic hepatitis C: Histological and virological correlations. J Viral Hepat.

[ppat-0020059-b034] Patel K, Zekry A, McHutchison JG (2005).

[ppat-0020059-b035] Hamamoto S, Uchida Y, Wada T, Moritani M, Sato S (2005). Changes in serum lipid concentrations in patients with chronic hepatitis C virus positive hepatitis responsive or non-responsive to interferon therapy. J Gastroenterol Hepatol.

[ppat-0020059-b036] Petit JM, Benichou M, Duvillard L, Jooste V, Bour JB (2003). Hepatitis C virus-associated hypobetalipoproteinemia is correlated with plasma viral load, steatosis, and liver fibrosis. Am J Gastroenterol.

[ppat-0020059-b037] Ye J, Wang C, Sumpter R, Brown MS, Goldstein JL (2003). Disruption of hepatitis C virus RNA replication through inhibition of host protein geranylgeranylation. Proc Natl Acad Sci U S A.

[ppat-0020059-b038] Wang C, Gale M, Keller BC, Huang H, Brown MS (2005). Identification of FBL2 as a geranylgeranylated cellular protein required for hepatitis C virus RNA replication. Mol Cell.

[ppat-0020059-b039] Horton JD, Shah NA, Warrington JA, Anderson NN, Park SW (2003). Combined analysis of oligonucleotide microarray data from transgenic and knockout mice identifies direct SREBP target genes. Proc Natl Acad Sci U S A.

[ppat-0020059-b040] Brown MS, Goldstein JL (1997). The SREBP pathway: Regulation of cholesterol metabolism by proteolysis of a membrane-bound transcription factor. Cell.

[ppat-0020059-b041] Brown AJ, Sun L, Feramisco JD, Brown MS, Goldstein JL (2002). Cholesterol addition to ER membranes alters conformation of SCAP, the SREBP escort protein that regulates cholesterol metabolism. Mol Cell.

[ppat-0020059-b042] Brown MS, Goldstein JL (1999). A proteolytic pathway that controls the cholesterol content of membranes, cells, and blood. Proc Natl Acad Sci U S A.

[ppat-0020059-b043] Jacobs JM, Diamond DL, Chan EY, Gritsenko MA, Qian W (2005). Proteome analysis of liver cells expressing a full-length hepatitis C virus (HCV) replicon and biopsy specimens of posttransplantation liver from HCV-infected patients. J Virol.

[ppat-0020059-b044] Loguercio C, Federico A (2003). Oxidative stress in viral and alcoholic hepatitis. Free Radic Biol Med.

[ppat-0020059-b045] Saleem N, Mubarik A, Qureshi AH, Siddiq M, Ahmad M (2004). Is there a correlation between degree of viremia and liver histology in chronic hepatitis C?. J Pak Med Assoc.

[ppat-0020059-b046] Boyacioglu S, Gur G, Yilmaz U, Korkmaz M, Demirhan B (2004). Investigation of possible clinical and laboratory predictors of liver fibrosis in hemodialysis patients infected with hepatitis C virus. Transplant Proc.

[ppat-0020059-b047] Garcia-Samaniego J, Soriano V, Castilla J, Bravo R, Moreno A (1997). Influence of hepatitis C virus genotypes and HIV infection on histological severity of chronic hepatitis C. The Hepatitis/HIV Spanish Study Group. Am J Gastroenterol.

[ppat-0020059-b048] Benhamou Y, Bochet M, Di MV, Charlotte F, Azria F (1999). Liver fibrosis progression in human immunodeficiency virus and hepatitis C virus coinfected patients. The Multivirc Group. Hepatology.

[ppat-0020059-b049] Soto B, Sanchez-Quijano A, Rodrigo L, del Olmo JA, Garcia-Bengoechea M (1997). Human immunodeficiency virus infection modifies the natural history of chronic parenterally-acquired hepatitis C with an unusually rapid progression to cirrhosis. J Hepatol.

[ppat-0020059-b050] Graham CS, Baden LR, Yu E, Mrus JM, Carnie J (2001). Influence of human immunodeficiency virus infection on the course of hepatitis C virus infection: A meta-analysis. Clin Infect Dis.

[ppat-0020059-b051] Berenguer M (2002). Natural history of recurrent hepatitis C. Liver Transpl.

[ppat-0020059-b052] Berenguer M, Prieto M, Rayon JM, Mora J, Pastor M (2000). Natural history of clinically compensated hepatitis C virus-related graft cirrhosis after liver transplantation. Hepatology.

[ppat-0020059-b053] Garcia-Retortillo M, Forns X, Feliu A, Moitinho E, Costa J (2002). Hepatitis C virus kinetics during and immediately after liver transplantation. Hepatology.

[ppat-0020059-b054] Pal S, Shuhart MC, Thomassen L, Emerson SS, Su T (2006). Intrahepatic hepatitis C virus replication correlates with chronic hepatitis C disease severity in vivo. J Virol.

[ppat-0020059-b055] Brazma A, Hingamp P, Quackenbush J, Sherlock G, Spellman P (2001). Minimum information about a microarray experiment (MIAME) toward standards for microarray data. Nat Genet.

[ppat-0020059-b056] Batts KP, Ludwig J (1995). Chronic hepatitis. An update on terminology and reporting. Am J Surg Pathol.

[ppat-0020059-b057] Mayerat C, Burgisser P, Lavanchy D, Mantegani A, Frei PC (1996). Comparison of a competitive combined reverse transcription-PCR assay with a branched-DNA assay for hepatitis C virus RNA quantitation. J Clin Microbiol.

[ppat-0020059-b058] Walters KA, Joyce MA, Thompson JC, Proll S, Wallace J (2006). Application of functional genomics to the chimeric mouse model of HCV infection: Optimization of microarray protocols and genomics analysis. Virology J.

